# Class merging degrades learned representations in potato-harvesting object detection despite operational equivalence

**DOI:** 10.3389/fpls.2026.1847038

**Published:** 2026-09-09

**Authors:** Joonam Kim, Rena Yoshitoshi, Giryeon Kim, Noriko Deguchi, Hideo Kainuma, Shinori Tsuchiya, Kenichi Tokuda

**Affiliations:** 1 Research Center for Agricultural Robotics, NARO, Tsukuba, Japan; 2 Hokkaido Agricultural Research Center, NARO, Memuro, Japan

**Keywords:** agricultural object detection, class hierarchy, fine-grained supervision, label granularity, label merging, potato harvester, precision agriculture, YOLOX

## Abstract

**Introduction:**

In artificial intelligence (AI)-operated mobile potato harvesting, separating field impurities (stones and soil clods) from potatoes determines product quality. Because both types trigger the same pneumatic removal action, merging them into one impurity class is a natural simplification; we found that it degrades detection quality in every random seed tested, relative to a 3-label formulation (potato vs. stone vs. soil clod).

**Methods:**

We trained YOLOX-Small under two label structures, with identical images, boxes, augmentation and hyper-parameters: 3-label (potato, stone, soil clod) and 2-label (potato, impurity). The models were fitted on 11,869 images of ten cultivars from seven Hokkaido farms collected up to the 2024 harvest — the training and validation splits of a 13,189-image corpus divided 7:2:1. The test set was a separate 2,960-image corpus from the 2025 season sharing no image or acquisition session with it (110,420 potato and 15,234 impurity instances; 12.1% prevalence). Each structure used five seeds. Both were scored on a common two-class target, potato versus impurity: the stone and soil-clod predictions of the 3-label model were pooled into a single impurity class before any metric was computed. Detection was assessed with potato misclassification rate (PMR), impurity detection rate (IDR), impurity precision and average precision (AP) over a 17×17 threshold grid; the 512-dimensional backbone representations were compared on the same target.

**Results:**

The 3-label structure achieved higher impurity precision than the 2-label structure at the default operating point (83.20 ± 0.85% vs. 70.07 ± 1.59%; +13.1 percentage points (pp); 95% confidence interval (CI) [10.5, 15.7]) and higher threshold-free impurity AP at 50% box overlap (79.8 ± 1.3% vs. 65.6 ± 0.8%; +14.2 pp), in every seed across the operational threshold region. The 2-label representation showed a 39% smaller centroid distance, 37% lower silhouette separability and 30–41% lower per-channel Fisher ratios. The advantage persisted although the subclass task failed asymmetrically: soil clods collapsed into stone (recall 1.84 ± 0.13%) while stones were correctly subclassed (80.69 ± 1.12%).

**Discussion:**

Fine-grained supervision appears to shape the backbone representation independently of subclass accuracy: operational equivalence does not entail representational equivalence. The link between representation and performance is observational, and all results come from one detector architecture and region.

## 1 Introduction

Vision-based automation in agriculture has evolved rapidly from handcrafted features to deep detectors (Ghazal et al., 2024; Badgujar et al., 2024). Beyond in-field detection, intelligent optical sensing is increasingly used for non-destructive assessment of agricultural-product quality and authenticity, for example visible/near-infrared imaging with explainable machine learning for geographical-origin discrimination (Zhang et al., 2026) and multi-class deep-learning identification of adulterated products (Ma et al., 2026); our task shares the same premise — that learned representations, not only nominal accuracy, determine field reliability. In potato harvesting, modern mobile harvesters excavate tubers and convey them past an inspection point where an AI vision system identifies each object. Pneumatic actuators then selectively deflect detected impurities — primarily stones and soil clods — off the conveyor while potatoes continue downstream. Among AI vision systems, the YOLO family dominates real-time applications owing to its single-stage design and favorable speed–accuracy trade-off (Alif and Hussain, 2024; Ge et al., 2021). Transformer-based detectors such as the detection transformer, DETR (Carion et al., 2020), have attracted attention, but their computational cost limits deployment on mobile edge devices. Recent studies on lightweight detectors for resource-constrained platforms (Rey et al., 2025; Ma et al., 2023) and on deep learning across the potato production chain (Wang and Su, 2024; Johnson and Auat Cheein, 2023) further support compact YOLO architectures for agricultural field equipment.

Our previous field trials demonstrated that a YOLOX-based system could sustain potato misclassification rates below 0.1% with impurity detection rates above 89% across ten cultivars and seven farms in Hokkaido (Kim et al., 2026), and a companion study compared the YOLOX and YOLOv12 architectures for edge deployment on Jetson AGX Orin hardware in the same harvesting context (Kim et al., 2025). Those studies addressed field deployment and edge latency; neither manipulated the label structure nor examined the learned representation. The present study is, to our knowledge, the first to isolate label structure as the sole training variable in an agricultural detector and to connect it to backbone representation. Potato harvesting adds domain-specific constraints: the detector must cope with fluctuating illumination and soil-covered, partly overlapping objects on a moving conveyor (Korchagin et al., 2021) and, on a harvester, with mechanical vibration and a class distribution skewed heavily toward potatoes (Kim et al., 2026).

A design question remains regarding how object classes should be structured. Stones and soil clods trigger the same removal action and are operationally interchangeable. Combining them into a single “impurity” label reduces the label set from three object classes to two, reduces annotation effort, and aligns the model output directly with the actuator logic. A simpler task would be expected to be easier to learn; however, evidence from other domains suggests the opposite. Convolutional neural networks (CNNs) trained with fine-grained labels almost always achieve higher coarse-class accuracy than those trained with coarse labels alone (Chen et al., 2018); learning with only coarse labels can cause fine-grained feature collapse (Bukchin et al., 2021); minority classes are learned less well under class imbalance (Buda et al., 2018; Cui et al., 2019); hierarchical or taxonomy-aware models exploit fine-grained structure to regularize shared representations (Deng et al., 2014; Yan et al., 2015; Silla and Freitas, 2011); and multitask learning shows that an auxiliary task can improve a primary one through the shared representation (Caruana, 1997; Ruder, 2017).

However, this evidence comes almost entirely from image-classification benchmarks. Our contribution is distinct in three concrete respects. First, we study a real one-stage agricultural object detector (YOLOX-Small), where localization, objectness and classification losses interact, rather than a pure classifier. Second, we frame the merge as operationally equivalent, because the two subclasses trigger an identical actuator and merging is therefore the intuitively “free” choice a practitioner makes, and we show that it is not free. Third, we characterize the effect with an exhaustive confidence-threshold grid and several complementary probes of the same backbone. We do not claim the phenomenon itself is novel; we claim the operational-equivalence framing and the detector-level, multi-probe evidence are.

**Figure 1:**
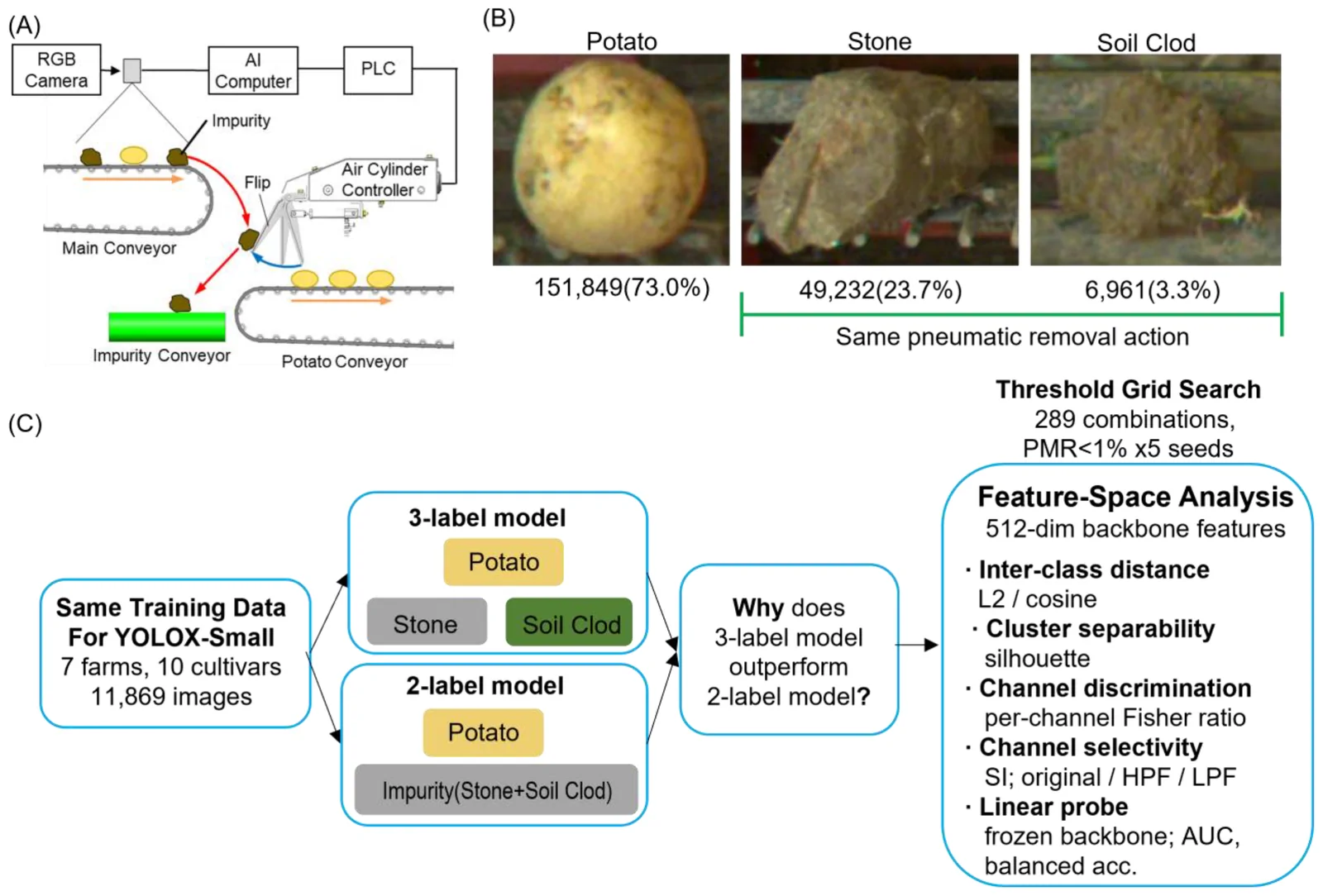
Overview of the AI-powered potato-harvesting system and experimental design. **(A)** Camera-based impurity removal on a mobile harvester: a red–green–blue (RGB) camera images objects on the conveyor, the AI computer classifies each object, and a programmable logic controller (PLC) drives the pneumatic actuators that deflect detected impurities. **(B)** The three object classes, shown as single-object excerpts cut from training images so that their appearance can be compared; the images themselves are full conveyor frames containing many annotated objects, not object-centered crops (**Supplementary Figure A1**). The figures beneath each panel are object instance counts over the training and validation splits. Stones and soil clods trigger the same pneumatic removal action despite distinct visual characteristics. **(C)** Experimental pipeline: both label structures are trained on identical data with only the label structure differing; a threshold grid search establishes performance dominance and feature-space probes provide converging evidence for the underlying representational difference. In **(C)**, SI is the per-channel selectivity index and HPF and LPF are the high-pass- and low-pass-filtered inputs defined in Section 2.5.

Field experience contradicted the expectation of simplicity: the binary models exhibited systematically lower detection precision across a wide range of confidence thresholds. This matters in practice because every false impurity detection fires a pneumatic finger, adding mechanical wear, wasting compressed air, and risking displacement of adjacent potatoes. Meanwhile the 3-label model largely fails to recover the minority soil-clod subclass internally yet maintains a tighter impurity–potato boundary. This mismatch between subclass accuracy and binary discrimination points to a representational effect of the label structure. **Figure 1** summarizes the harvesting system, the three object classes, and the experimental design used to investigate it.

To probe the mechanism, we drew on established feature-space tools: t-distributed stochastic neighbor embedding (t-SNE; van der Maaten and Hinton, 2008) and principal component analysis (PCA) for visualization, silhouette analysis (Rousseeuw, 1987) and Fisher ratios (Fisher, 1936) for cluster quality, and channel-selectivity indices inspired by neuronal selectivity (Zhou et al., 2015; Bau et al., 2017). We additionally use a frozen-backbone linear probe, which removes the detection head and asks directly how linearly separable potato and impurity are in the learned features. Other tools exist for related questions, such as Grad-CAM for spatial attribution (Selvaraju et al., 2017), representational similarity analysis for comparing distance structures across models (Kriegeskorte et al., 2008), and Fourier analysis of channel activation for identifying the spatial-frequency scales a network relies on (Yin et al., 2019); the last of these motivates the frequency decomposition reported in Section 3.3. Work on neural collapse describes the terminal geometry of trained classifiers (Papyan et al., 2020; Zhu et al., 2021), and unequal class frequencies are known to distort that geometry (Fang et al., 2021). Related work shows that even when coarse labels drive representations toward apparent collapse, the learned features retain fine-grained subclass structure that unsupervised clustering can recover (Yang et al., 2023). Merging classes of very different frequency may therefore disturb feature geometry while leaving subclass information partly latent.

## 2 Methods

This section describes the data, the two label structures, and the measurements used to compare them. The design isolates label structure as the only experimental variable: the same images, bounding boxes, augmentation, hyper-parameters and random seeds are used for both variants, and only the class labels differ.

### 2.1 Dataset

Two disjoint corpora were used: the 2024 corpus, from which the training, validation and held-out splits are drawn, and a separate 2025 test corpus. Both were re-audited for the present study to guarantee separation at the acquisition-session level and in time. The two corpora were collected in different harvest seasons, so the split is temporal rather than farm-based; we state below exactly what does and does not overlap. **Table 1** lists every split with its role, its size and its share of the data.

**Table 1:** The data splits, with the role and size of each.

Split	Role	Images	Share of its corpus	Overlap with test set (images)
2024 training split	Weight updates	9,232	70%	0
2024 validation split	Watched during training	2,637	20%	0
2024 held-out split	Held out by the same division; not used in this study	1,320	10%	0
2024 corpus (total)	Training + validation = 11,869 images	13,189	100%	0
2025 test set	Every result reported in this paper	2,960	separate corpus	—

The 2024 corpus was divided once in the ratio 7:2:1; the 2025 corpus is a separate test set and supplies every result reported here. Over both corpora (16,149 images) the shares are 57.2% training, 16.3% validation, 8.2% held out and 18.3% test. Both label structures use exactly the same splits, the same images and the same boxes; only the class labels attached to those boxes differ. Overlap is reported against the 2,960-image test set.

Training data. The images used for training were collected from seven commercial potato farms and ten cultivars in Hokkaido, Japan, during harvest seasons up to 2024. That 2024 corpus of 13,189 images was divided once, in the ratio 7:2:1, into 9,232 images for weight updates, 2,637 for monitoring during training, and 1,320 held out. The division is conventional, was fixed when the corpus was assembled and before any experiment reported here and was applied identically to both label structures and to all five seeds. We do not evaluate on the held-out split: it belongs to the 2024 corpus on which our detectors were developed, and a result measured on it would leave open the possibility that the effect is a property of that particular collection — which is precisely what the season-separated 2025 corpus is used to exclude. The training and validation splits together are the 11,869 images on which the models were fitted. Those 11,869 images contain 208,042 object instances: 151,849 potatoes (73.0%), 49,232 stones (23.7%), and 6,961 soil clods (3.3%). For the 2-label variant, stones and soil clods were merged into a single impurity class (56,193 instances, 27.0%), giving a potato-to-impurity ratio of approximately 2.7:1. Both label structures were trained on the same images with the same bounding boxes; only the class labels differed. The low soil-clod proportion reflects natural field conditions, as soil aggregates frequently break apart during mechanical excavation and conveyor transport, and it produces a stone-to-soil-clod ratio of about 7:1 within the impurity category — stones account for 87.6% of all impurity instances. This imbalance is central to the mechanism considered in Section 4.2.

Test data. A separate test set of 2,960 images was collected during the 2025 harvest season, one full season after the latest training data. Its images come from 896 acquisition sessions, a session being one continuous recording of the conveyor camera, identified by the file-name prefix that each recording carries; session sizes range from a single image to 410, so images within a session are consecutive frames and can be near-duplicates. It spans seven commercial farms and seven cultivars and contains 110,420 potato instances and 15,234 impurity instances (10,468 stones and 4,766 soil clods), an observed impurity prevalence of 12.1%. This set was originally assembled during routine field operation to accumulate cases that the deployed detector handled poorly, with a view to further model improvement; because the deployed system already met its operational targets, the set was repurposed as the test set here. It is therefore deliberately enriched both in impurities and in difficult cases and is harder than the field average by construction. Annotation was performed with a semi-automatic labeling tool, and every label was subsequently verified and corrected by a human annotator. Representative full frames from both corpora are shown side by side in **Supplementary Figure A1**.

Two consequences follow and we state them explicitly. First, absolute IDR and precision measured on this set are expected to be lower than field-average performance and should not be read as deployment estimates; the field figures reported previously (Kim et al., 2026) remain the appropriate reference for that. Second, because both label structures are evaluated on exactly the same images with exactly the same bounding boxes, the comparison between them is paired and no part of it can be attributed to a difference in test material. Recall-based quantities such as IDR are invariant to the overall impurity prevalence only if the conditional subtype and difficulty distributions are held fixed, which the accumulation rule does not guarantee. Precision depends on prevalence directly, so the precision gap is re-weighted to lower, field-realistic impurity rates in Section 3.2 rather than being assumed invariant. Under that prevalence-only model the gap grows as impurities become rarer, which would mean the enriched set understates it; this is a statement about prevalence alone and does not fix the direction of the total selection effect, because subtype and difficulty composition also changed. Soil clods constitute 31.3% of test-set impurity instances against 12.4% in training, because difficulty-based accumulation preferentially retained the subtype the deployed detector handled least reliably. The 7:1 training imbalance, not this test-set ratio, is what drives the mechanism considered in Section 4.2.

The detector that accumulated those difficult cases was itself a 3-label model, since that is the label structure deployed in our production system. Because test-set cases were accumulated conditional on the behavior of a deployed 3-label detector, the absolute metrics reported here should not be read as unbiased estimates of field-wide performance. Both models are scored on the same images and the same boxes, so no part of the comparison is attributable to a difference in test material; but selection conditional on prior 3-label behavior may still affect the estimated difference between the two, and its direction cannot be determined from the selection rule available to us. We return to this in Section 4.4.

Split audit. The 2024 corpus of 13,189 images was divided once, in the ratio 7:2:1, into a training split of 9,232 images (70.0%), a validation split of 2,637 (20.0%) and a held-out split of 1,320 (10.0%); the training and validation splits together are the 11,869 images described above. The 2025 corpus of 2,960 images is a separate test set and is the source of every result reported in this paper. Over both corpora, 16,149 images in total, the shares are 57.2% training, 16.3% validation, 8.2% held out and 18.3% test. The two corpora share 0 images and 0 acquisition sessions, and the 2024 held-out split likewise shares none with the 2025 corpus (**Table 1**). The 7:2:1 division was made at the image level. It does not bear on the comparison, because the validation split was only monitored and every reported result comes from the 2025 corpus. The two corpora come from different harvest seasons: training up to 2024, test in 2025. No video sequence or acquisition session can therefore appear in both, and the separation is temporal rather than incidental. Farms and cultivars do recur across the two corpora, so the design is a temporal holdout and not a farm-level holdout. The test set is drawn from the same commercial operations in the following season, which is the condition a deployed detector faces. As a robustness check on farm composition, we also re-computed the comparison after excluding two farms from the test set (Section 3.1 and **Supplementary Table A1**).

### 2.2 Model and training

This subsection states the detector, the training protocol, and the replication scheme. Everything reported here is identical between the two label structures; the only difference is the set of class labels attached to the same bounding boxes.

**Figure 2:**
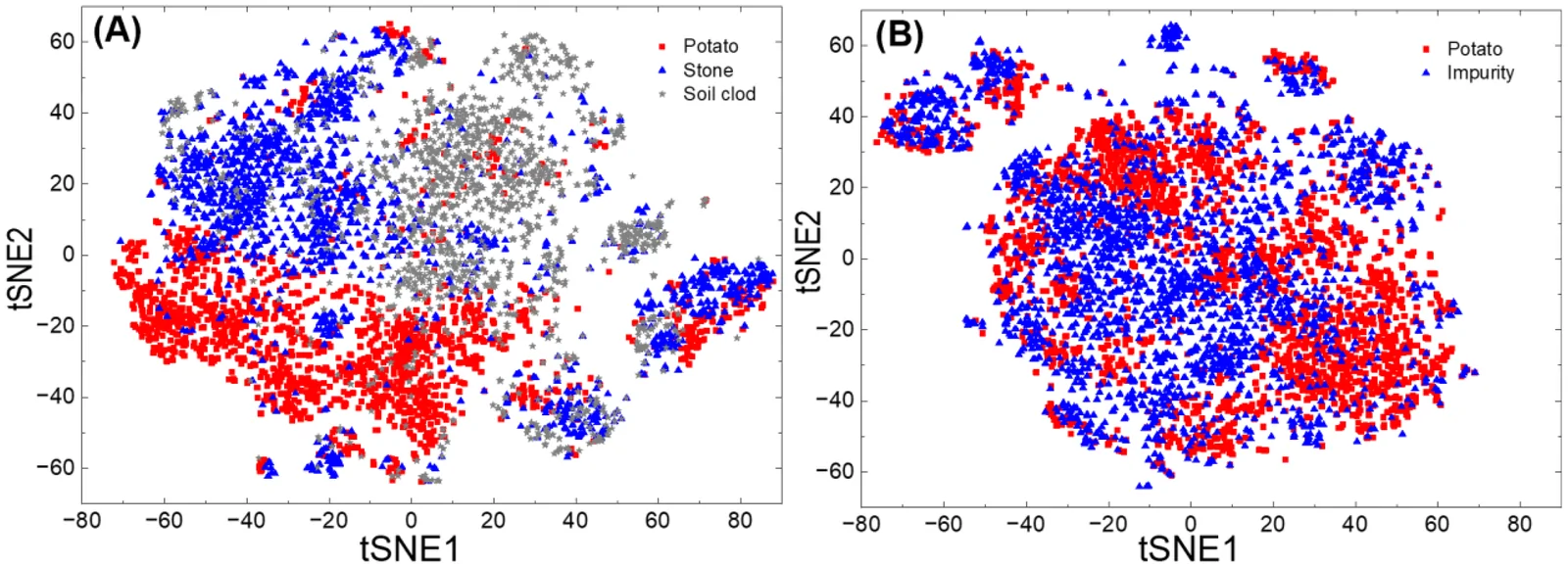
t-SNE projections of the 512-dimensional backbone features for a representative seed (seed 42; class-balanced subsample of about 5,000 objects). **(A)** 3-label model: potatoes and stones form largely separable regions and soil clods embed adjacent to stones rather than dispersing into the potato distribution. **(B)** 2-label model: impurity samples scatter across the potato distribution and cluster coherence is visibly reduced.

**Figure 3:**
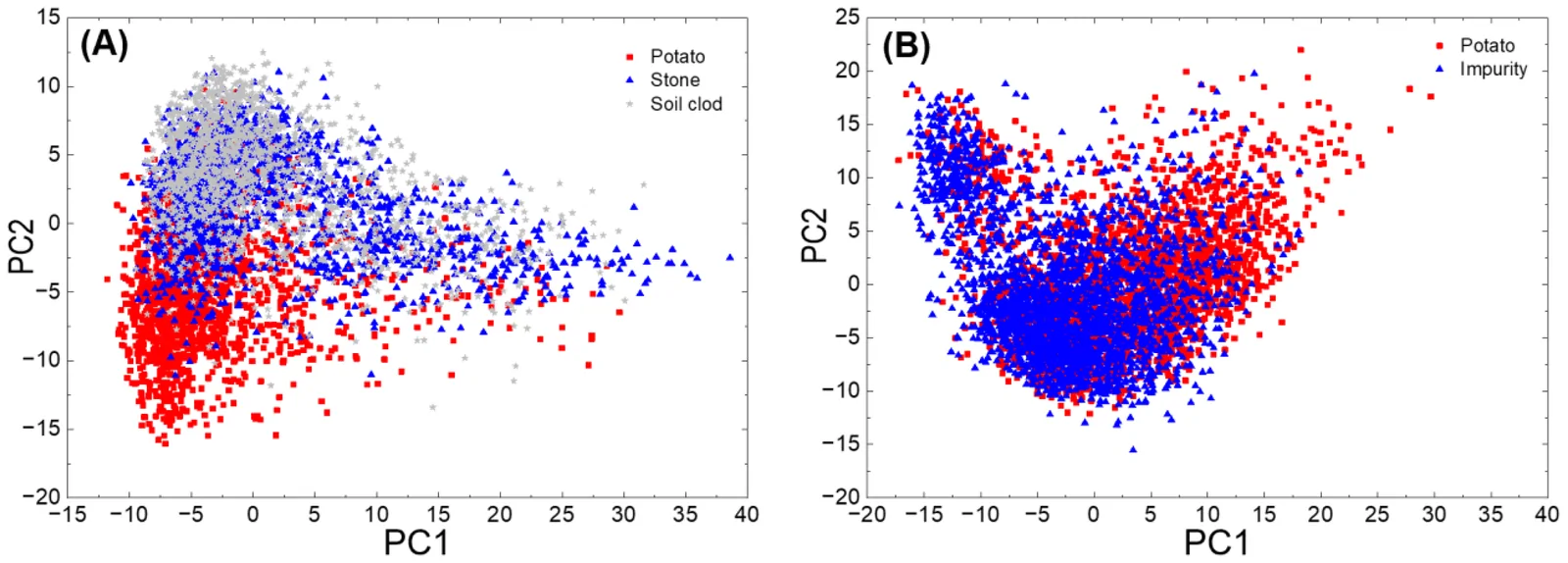
Principal-component projections (first two components) of the same features and seed as **Figure 2**. **(A)** 3-label model. **(B)** 2-label model, in which the classes overlap more extensively. The first two components account for 12.3% and 5.8% of the total variance in the 3-label model and 10.5% and 7.9% in the 2-label model.

We used YOLOX-Small (Megvii-BaseDetection, 2022) for its Apache-2.0 license, anchor-free head, and demonstrated efficiency on Jetson AGX Orin hardware (Kim et al., 2025; Rey et al., 2025). Two variants were trained: a 3-label model (labels potato, stone, soil clod) and a 2-label model (labels potato, impurity). All training settings were identical between the two variants and across seeds: 250 epochs, stochastic gradient descent (SGD) with momentum 0.9, initial learning rate 0.01 with cosine annealing, weight decay 5×10^−4^, batch size 16, and mosaic augmentation (Bochkovskiy et al., 2020) with mixup and random hue–saturation–value (HSV) transforms (Shorten and Khoshgoftaar, 2019). The backbone, neck, training schedule and all configured hyper-parameters were therefore held constant, while the classification output dimensionality and the target labels differed. The 250-epoch schedule was fixed in advance and applied identically to both label structures and all seeds. Weight updates used the 9,232-image training split; the 2,637-image validation split was watched during training but was never used to pick a checkpoint, because the final epoch was taken in every run (Section 2.1). No hyper-parameter search was performed and the 2025 test set played no part in any training decision. Mosaic augmentation is disabled over the closing epochs of the schedule, which is where the operational metrics reach their final level. To distinguish the label-structure effect from initialization variance, each variant was trained under five random seeds (42, 123, 777, 2026, 31415), giving ten trained models, five per label structure. Every result in Section 3 is a mean ± standard deviation over these five seeds, with the two label structures compared pairwise by seed; no single-seed value is reported anywhere except the qualitative t-SNE and PCA projections of **Figures 2** and **3**, which are drawn from seed 42.

### 2.3 Evaluation metrics

This subsection defines the metrics used throughout and the operational targets attached to them. The first four are the deployment metrics of our production system; the last two are threshold-free measures. We adopt the operational metrics defined in our deployment study (Kim et al., 2026):

PMR is the fraction of potato instances erroneously predicted as impurity: PMR = N_potato→impurity_/N_potato,total_. The operational target was a PMR of < 1%.

IDR is the recall of impurity detection: IDR = N_impurity,correct_/N_impurity,total_. Thus, the IDR is equivalent to impurity recall. For the 3-label model, cross-impurity confusion (stone identified as soil clod, or vice versa) counted as correct because both trigger the same removal action. The operational target was an IDR of > 60%.

Impurity-to-potato confusion rate (ICR, reported as IMR in our deployment study) is the fraction of impurity instances erroneously classified as potato: ICR = N_impurity→potato_/N_impurity,total_. These impurities are not removed but pass through. ICR counts only impurities that the detector localized and then labeled potato. Impurities it never localized appear in the denominator of both ICR and IDR but in neither numerator. The share of impurities left unremoved for any reason is therefore 1 − IDR — 26.3% for 3-label and 35.4% for 2-label at the default threshold — and ICR is the part of that share caused by misclassification rather than by missed localization.

Impurity precision is the fraction of impurity class detections that correspond to true impurities: precision = N_impurity,correct_/N_impurity,predicted_. For the 3-label model the stone and soil-clod predictions are pooled into a single impurity class before this quantity is computed, exactly as they are for IDR and for AP, so that both label structures are scored on the same two-class target and the two precisions are like-for-like. Potato precision is reported separately in **Table 2**. False impurity predictions cause unnecessary pneumatic actuations; therefore, precision reflects operational efficiency. We also report the harmonic mean of the IDR and impurity precision, F1 = 2 × IDR × precision/(IDR + precision).

**Table 2:** Per-class precision, recall and F1 at threshold 0.5 (potato and merged impurity; mean ± SD over five seeds).

Model	Class	Precision (%)	Recall (%)	F1 (%)
3-label	Potato	98.88 ± 0.16	95.80 ± 0.37	97.32 ± 0.22
3-label	Impurity	83.20 ± 0.85	73.74 ± 1.68	78.17 ± 0.93
2-label	Potato	97.40 ± 0.18	98.17 ± 0.16	97.78 ± 0.08
2-label	Impurity	70.07 ± 1.59	64.56 ± 1.79	67.17 ± 0.75

Potato precision is computed on matched detections while impurity precision includes background detections, so the two rows of the precision column are not on a common denominator (see footnote).

Impurity precision is a detection-level quantity: its denominator is every impurity-class prediction, including background detections that match no ground-truth object, because each of those fires an actuator. Potato precision is a confusion-level quantity restricted to matched detections, since a background detection labeled potato triggers no action. The two are therefore not on the same denominator and should not be compared with each other. For completeness, potato precision computed on the impurity-class denominator, that is including background detections labeled potato, is 95.09 ± 0.23% for 3-label and 78.88 ± 3.44% for 2-label; the direction of the comparison between label structures is unchanged under either convention.

Threshold-free detection quality is additionally reported as average precision at an intersection-over-union (IoU) of 0.50 (AP50) and averaged over IoU 0.50 to 0.95 (AP50:95). AP is computed both per native class and on a merged two-class-equivalent task, in which the stone and soil-clod predictions of the 3-label model are pooled into a single impurity class, so that the two label structures are scored on identical targets.

### 2.4 Threshold grid and detection matching

This subsection defines the operating points at which the two models are compared and the rule by which a prediction is counted as correct. Both are stated in full because the comparison would otherwise depend on unstated choices.

To verify that performance differences were not threshold artifacts, we swept both the potato and impurity confidence thresholds from 0.10 to 0.90 in steps of 0.05 (17 × 17 = 289 operating points per model), computing all metrics from cached raw predictions. We restricted attention to the feasible region (PMR < 1%) and, more conservatively, to an operational region (PMR < 1% and IDR ≥ 60% for both models). Detections were matched to ground truth by a fixed protocol: the fused detection score is objectness × class probability; per-class confidence filtering is applied; class-wise non-maximum suppression (NMS) uses IoU 0.5; detections are matched greedily one-to-one in descending confidence at match-IoU 0.5; duplicate detections on an already-matched box and background detections are counted as false positives, and unmatched ground-truth boxes as misses. NMS and matching are performed on each model’s native classes, and the 3-label model’s stone and soil-clod matches are mapped to impurity only afterwards, when the metric is aggregated; scores are never merged before suppression. Because NMS is class-wise, the 3-label model can in principle leave both a stone box and a soil-clod box on one object, and the unmatched one is then counted as a duplicate false positive. This works against rather than for the 3-label model, and in practice it produces fewer such duplicates than the 2-label model (325 against 942; **Table 3**). **Table 3** reports the resulting matching diagnostics, and Section 3.2 reports sensitivity to the match-IoU threshold.

**Table 3:** Detection-matching diagnostics at the default threshold (0.5, 0.5), mean over five seeds.

Quantity	3-label	2-label
Candidates after NMS	140,068	289,385
Selected at default threshold	125,077	152,629
Matched one-to-one	118,806	121,457
Class-confused matches	4,489	3,219
Duplicate detections (false positive)	325	942
Background false positives (total)	5,946	30,231
of which predicted impurity	1,678	3,889
of which predicted potato	4,267	26,343
Unmatched ground truth (miss)	6,848	4,197

Counts are seed-averaged and rounded, so row and column totals may differ by one. The 2-label model emits about five times as many background false positives overall, and 2.3 times as many of the impurity-class ones that enter impurity precision (3,889 vs 1,678). Potato-to-impurity confusion in fact falls under merging (592 vs 322, **Table 6**), so the precision loss comes from background detections labeled impurity rather than from confused potatoes.

Prevalence sensitivity. Because the test set is enriched in impurities, impurity precision is additionally recomputed under hypothetical impurity prevalences using a prior-shift transformation. Writing P_0_ for the precision observed at the test-set prevalence p_0_ = 0.121 and p for the hypothetical prevalence, the re-weighted precision is P(p) = P_0_·r/[P_0_·r + (1 − P_0_)], with r = [p/(1−p)] ÷ [p_0_/(1−p_0_)]. The transformation assumes that the number of false-positive opportunities scales with the number of non-impurity objects, and that the conditional distributions of impurity subtype and case difficulty, and therefore each model’s per-object detection and false-positive behavior, are unchanged as prevalence varies. In object detection the first assumption is only approximate, since background false positives also depend on image count, background area and object density rather than on negative-object count alone. The result is therefore a hypothetical prior-shift analysis of one variable and not a field precision estimate.

Subtype-composition sensitivity. The test set is also enriched in soil clods relative to training, and the two label structures do not differ equally on the two impurity subtypes, so the subtype mix is a second respect in which the test set departs from field conditions. We therefore recompute the comparison with the impurity population re-weighted to the training subtype composition, holding the total number of impurity instances fixed. Each subtype keeps its own measured per-instance detection rate, and two error sources are held at their observed per-seed values: the number of potatoes misclassified as impurity, and the number of background detections labeled impurity. Both are counted over the potato population and the image background rather than over the impurity population, so they are approximately, though not exactly, unaffected by a change in the ratio of stones to soil clods; this is the stronger of the two assumptions. The two corrections are also applied together, in which case the subtype re-weighting is performed first and the prevalence transformation is applied to the result. Both are sensitivity calculations on a single variable and neither is a field estimate.

### 2.5 Feature-space analysis

This subsection describes how the backbone representations were extracted and the five ways they were analyzed. The extraction is deliberately minimal so that the comparison reflects the learned features rather than any post-processing choice.

For every ground-truth object we placed a forward hook on backbone.backbone.dark5 and read the single 512-dimensional activation vector at the feature-map cell containing the box center — with no pooling and no normalization. Extraction is performed once per image during a standard forward pass and does not depend on any detection confidence threshold. Because these features precede the detection head, they reflect the learned representation underlying all subsequent decisions. We analyzed them five ways: (i) inter-class L2 and cosine distances between class centroids; (ii) cluster separability via silhouette scores; (iii) per-channel Fisher discriminant ratios; (iv) a per-channel selectivity index; and (v) a frozen-backbone linear probe — a logistic-regression classifier trained on the extracted features to separate potato from impurity, with the detection head removed. The probe splits the test set at the image level, not the object level: the 2,957 images that contain objects are permuted once with a fixed seed and the first 30% (887 images) are held out, which yields 88,174 training and 37,480 held-out objects. The same split is applied to both label structures and to all five model seeds, so the two models are compared on identical training and test objects. Features are standardized, class weighting is inverse to frequency, and the logistic regression uses L2 regularization with C = 1.0 and a 2,000-iteration limit. Because images are disjoint across the split, no object shares an image with an object on the other side; acquisition sessions are not disjoint, however, so images captured seconds apart within one session can fall on opposite sides, and the absolute AUC should be read as an optimistic estimate of generalization to an unseen session. Because within-session similarity may affect the two representations to different degrees, its effect on the between-model gap cannot be assumed to cancel. We therefore also repeated the probe with the split taken over acquisition sessions rather than images, holding out 269 of the 896 sessions (95,279 training and 30,375 held-out objects) under otherwise identical settings. We report both and regard the session-disjoint version as the more conservative analysis. The probe is evaluated by the area under the receiver operating characteristic curve (AUC) and by balanced accuracy, and is swept over training-set sizes from 50 to 5,000 samples per class with five repeats per size. Two-dimensional t-SNE and PCA projections are additionally used for qualitative visualization.

The per-channel Fisher discriminant ratio is computed for each channel c as F_c_ = (μ_a_ − μ_b_)^2^/(σ^2a^ + σ^2b^), where μ and σ² are the mean and variance of that channel’s activation over the objects of each class. The primary comparison uses an identical potato-versus-impurity partition for both models: in the 3-label model the stone and soil-clod objects are pooled into one impurity class before the ratio is computed, so the two models are described on the same two categories with the same objects. As a secondary, descriptive result we also report the 3-label model on its two native subclass pairs (potato versus stone, potato versus soil clod), which the 2-label model cannot express; these native curves are not used for any between-model comparison. The per-channel selectivity index is SI = (μ_potato_ − μ_impurity_)/(μ_potato_ + μ_impurity_), and we report the median |SI| across the 512 channels. To ask whether any selectivity difference is confined to a particular spatial-frequency band, SI was computed three times: on the unfiltered images, on high-pass-filtered images (texture-dominant), and on low-pass-filtered images (shape-dominant), with the filtered image sets generated once and passed through the same forward hook.

Statistical analysis. With five seeds per label structure the comparison is paired by seed. We report the mean difference with a 95% confidence interval, Cohen’s d_z_ for paired effect size, and — because rank tests on n = 5 reach their floor at p = 0.0625 — effect sizes alongside the sign and Wilcoxon results, so that the reader can see both the effect size and the limits of significance testing at this sample size. Confidence intervals on the per-seed precision gap were obtained by bootstrap resampling of the matched detections (10,000 resamples; objects within an image are not independent, so these intervals are optimistic and are reported as within-seed sampling uncertainty only). All statistics were computed on the five paired seed-level values, never on individual threshold-grid points, which are not independent.

## 3 Results

This section reports the comparison in three steps. We first establish detection performance at the default operating point and its consistency across seeds, then show that the difference survives an exhaustive sweep of both confidence thresholds, and finally examine the backbone representations that accompany it.

### 3.1 Detection performance

This subsection establishes the primary result at the default operating point and its robustness across seeds. We first report the operational metrics, then a threshold-free average-precision comparison, then the confusion structure that explains where the difference arises. **Table 4** reports detection performance at the default confidence threshold of 0.5, averaged over five seeds.

**Table 4:** Detection performance at confidence threshold 0.5 (mean ± SD over five seeds).

Metric	3-label	2-label	3-label − 2-label (pp; final row = instances)
PMR (%)	0.54 ± 0.06	0.29 ± 0.03	+0.25
IDR (%)	73.74 ± 1.68	64.56 ± 1.79	+9.18
ICR (%)	7.86 ± 1.12	19.01 ± 1.37	−11.15
Impurity precision (%)	83.20 ± 0.85	70.07 ± 1.59	+13.13
IDR–precision F1 (%)	78.17 ± 0.93	67.17 ± 0.75	+11.00
Impurity correct/total	11,234 ± 255/15,234	9,835 ± 272/15,234	+1,399

Both models satisfied PMR < 1% (3-label 0.54 ± 0.06%, 2-label 0.29 ± 0.03%). The 3-label model detected more impurities (IDR 73.74 ± 1.68% vs. 64.56 ± 1.79%), had a much lower impurity-to-potato confusion rate (ICR 7.86 ± 1.12% vs. 19.01 ± 1.37%), and higher impurity precision (83.20 ± 0.85% vs. 70.07 ± 1.59%). The precision advantage is 13.13 pp (95% CI [10.54, 15.71]; Cohen’s d_z_ = 6.30; the two sets of five values do not overlap); with n = 5 the sign and Wilcoxon tests are at their floor (p = 0.0625) and are reported for completeness rather than relied upon. The advantage is also visible in threshold-free average precision (**Table 5**).

**Table 5:** Average precision (%) on two-class-equivalent targets (mean ± SD over five seeds).

AP metric	3-label	2-label	Δ (pp)
mAP50 (2-class-equivalent)	88.42 ± 0.81	80.86 ± 0.57	+7.56
mAP50:95 (2-class-equivalent)	80.04 ± 0.64	67.49 ± 0.87	+12.55
AP50 impurity (merged)	79.76 ± 1.30	65.59 ± 0.83	+14.18
AP50:95 impurity (merged)	67.43 ± 1.11	49.92 ± 0.69	+17.51
AP50 potato	97.08 ± 0.43	96.13 ± 0.44	+0.95
AP50:95 potato	92.65 ± 0.26	85.06 ± 1.22	+7.59

Merged-impurity AP50 is 79.8 ± 1.3% for 3-label versus 65.6 ± 0.8% for 2-label (+14.18 ± 1.60 pp; 95% CI [12.19, 16.17]; d_z_ = 8.86), and AP50:95 is 67.4 ± 1.1% versus 49.9 ± 0.7%. The potato class is essentially unaffected at IoU 0.50 (+0.95 pp) but is affected at stricter overlap: AP50:95 for potato is 92.65 ± 0.26% for 3-label against 85.06 ± 1.22% for 2-label (+7.59 pp). This is consistent with looser potato localization under merging, though it does not isolate it: the 2-label model returns slightly more potato detections (recall 98.17 ± 0.16% versus 95.80 ± 0.37%, **Table 2**) but with poorer box quality, so its advantage disappears at stricter overlap. **Table 2** gives per-class precision and recall on the same targets.

As a robustness check on farm composition, we recomputed the comparison after excluding two of the seven test-set farms, leaving 2,325 images. The precision advantage is essentially unchanged (2-label 71.05 ± 1.70% vs. 3-label 83.78 ± 0.98%; gap 12.73 pp), so the pooled estimate is not driven by that particular pair of sites. A per-folder breakdown supports this more directly. The test set is partitioned into fourteen folders, seven named by farm and seven by cultivar, and the precision advantage is positive in all seven farm-named folders, from +1.58 ± 5.21 pp to +23.69 ± 6.39 pp, and in six of the seven cultivar-named folders (**Supplementary Table A3**). Each folder therefore favors the 3-label model on its own. We stop short of claiming that removing any one folder would leave the pooled result unchanged: pooled precision is a ratio of summed counts rather than an average of folder-level rates, and the pooled figure was not recomputed folder by folder. Nor is this a leave-one-farm-out analysis, since the images in the cultivar-named folders cannot be attributed to individual farms in our records. The magnitude of the advantage also varies substantially across folders (Section 4.4). **Table 6** reports the confusion structure at threshold 0.5.

**Table 6:** Confusion structure at confidence threshold 0.5, restricted to ground-truth objects the model localized (mean counts over five seeds).

Ground truth	3-label localized	3-label → potato	3-label → impurity	2-label localized	2-label → potato	2-label → impurity
Potato	106,375	105,783	592	108,726	108,403	322
Soil clod	3,698	968 (26.2%)	2,730	3,793	2,085 (55.0%)	1,708
Stone	8,733	230 (2.6%)	8,503	8,938	811 (9.1%)	8,127

Percentages, shown for the two impurity rows, give the share of localized objects of that class that the model assigned to the potato class.

The 3-label → impurity column pools the stone and soil-clod predictions of the 3-label model; within it the model assigns 88 of the localized soil clods to the soil-clod class and 2,643 to the stone class, and 8,447 of the localized stones to the stone class. Merging roughly doubles the share of localized soil clods returned to the potato class (26.2% → 55.0%) and more than triples it for stones (2.6% → 9.1%).Counts are seed-averaged and rounded, so a row may not sum exactly.

Of the soil clods it localizes, the 3-label model returns 26.2% to the potato class and assigns 71.5% to the stone class, leaving 2.4% in the correct subclass (soil-clod subclass recall 1.84 ± 0.13% of all soil-clod instances). Yet its overall impurity detection is higher than that of the 2-label model. The subclass failure is asymmetric: stones are correctly subclassed (80.69 ± 1.12% of all stone instances, 96.7% of those localized) while the minority soil-clod subclass collapses into stone. Subclass separation therefore fails only for the minority subtype, while the potato–impurity boundary tightens. The instance-level reason for the recall difference is visible in **Table 6**: merging pushes a large share of localized impurities, soil clods above all, back across the potato boundary (26.2% to 55.0% for soil clods, 2.6% to 9.1% for stones), and that is where the IDR loss comes from. The precision loss has a different source. Boundary crossings remove an object from both the numerator and the denominator of precision, so they move it only slightly; the larger term is background detections labeled impurity, of which the 2-label model produces 3,889 against 1,678 (**Table 3**), while potato-to-impurity confusion falls under merging.

### 3.2 Threshold-independent dominance

**Figure 4:**
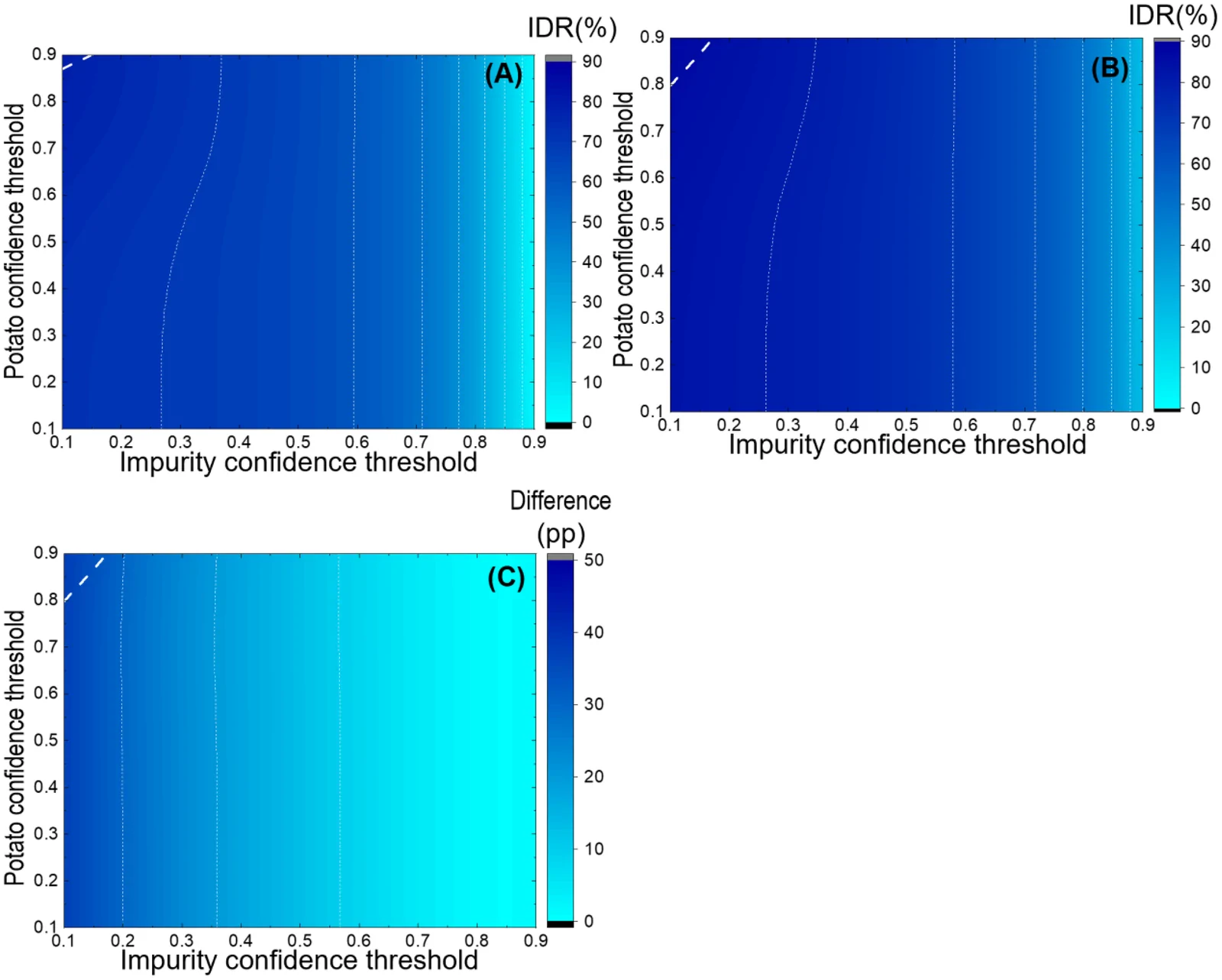
Impurity detection rate (%) across the 17 × 17 confidence-threshold grid, averaged over five seeds. **(A)** 3-label model and **(B)** 2-label model, sharing a common color scale. **(C)** Difference in impurity precision between the two label structures (3-label minus 2-label, percentage points) on the seed-averaged grid; the scale is strictly positive, so seed-averaged 3-label precision is higher at all 289 points. Per-seed agreement is lower (272 of 289 points; Section 3.2). In all panels the horizontal axis is the impurity confidence threshold, the vertical axis the potato confidence threshold, and the dashed line marks the PMR = 1% boundary of the 3-label model, with the feasible region below it. IDR is governed predominantly by the impurity threshold: averaged over the grid, sweeping the potato threshold across its full range changes IDR by 1.2 pp (3-label) and 1.7 pp (2-label), whereas sweeping the impurity threshold changes it by 61.8 pp and 71.4 pp. The residual dependence on the potato threshold is confined to low impurity thresholds, where weak impurity detections still compete with potato detections for the same ground-truth boxes — at an impurity threshold of 0.10 it reaches 4.2 pp (3-label) and 6.8 pp (2-label) — and falls below 0.1 pp at impurity thresholds of 0.65 and above, which is why the contours bend at the left of each panel and straighten toward the right. Only PMR varies appreciably along the vertical axis, which is why the feasibility boundary appears solely in the upper-left corner.

Because a single operating point could favor either model by chance, this subsection asks whether the 3-label advantage survives every other reasonable choice of thresholds, and across seeds. Two threshold regions are reported separately, because they answer different questions and give different numbers. Feasibility itself is not the discriminating property: both label structures satisfy PMR < 1% across nearly the entire grid — 2-label at 288 of 289 points in every seed, 3-label at between 261 and 288 points depending on the seed. The 2-label model is in fact the more conservative at the default operating point (PMR 0.29 ± 0.03% vs. 0.54 ± 0.06%), because merging pushes borderline objects toward the potato class and therefore produces fewer potato-to-impurity errors. The cost appears on the other side of the boundary: far more impurities are classified as potato (ICR 19.0% vs. 7.9%). What separates the two models is therefore precision within the feasible region, not the size of that region. **Figure 4** maps impurity detection rate across the grid for both label structures (panels a and b) and the impurity-precision difference between them (panel c).

Dominance across seeds. Within the operational region (PMR < 1% and IDR ≥ 60% for both models) the 3-label model has higher impurity precision at 849/849 points pooled over the five seeds (5/5 seed-pairs at 100%). Across all jointly feasible points (PMR < 1%) it wins at 1,376/1,410 (97.6%). On the seed-averaged grid the impurity-precision difference is positive at all 289 points, ranging from 0.7 to 38.0 pp, and it is positive in all five seeds at 272 of 289 points; the 17 points at which the seeds disagree all lie in a single impurity-threshold column (0.85) at which 2-label impurity recall has fallen to about 20%, far below the operational target, so none of the 849 operational points lie there. We report both rates with their region definitions rather than a single dominance figure; the seed-level comparison and the threshold-free AP in Section 3.1 carry the conclusion without relying on non-independent grid points.

Robustness. The precision gap is positive at every matching IoU and in every seed (12.80, 13.13 and 16.28 pp at IoU 0.30/0.50/0.75; **Supplementary Table A2**), growing to 16.28 pp at IoU 0.75, and it widens rather than narrows as impurity prevalence falls toward field-realistic rates. Because the test set is impurity-enriched relative to the field, we re-weighted precision to field-realistic impurity rates (**Table 7**).

**Table 7:** Sensitivity of impurity precision to impurity prevalence and to impurity subtype composition (mean ± SD over five seeds; the gap is the paired seed-level difference with its 95% confidence interval).

Scenario	3-label precision (%)	2-label precision (%)	Gap (pp)	95% CI (pp)
Observed subtype composition (soil clods 31.3% of impurity instances)				
12.1% prevalence (observed)	83.20 ± 0.85	70.07 ± 1.59	13.13	[10.54, 15.71]
10% prevalence	79.95 ± 0.98	65.35 ± 1.72	14.60	[11.76, 17.45]
7.5% prevalence	74.43 ± 1.16	57.93 ± 1.85	16.50	[13.34, 19.67]
5% prevalence	65.40 ± 1.38	47.20 ± 1.89	18.19	[14.77, 21.61]
Training subtype composition (soil clods 12.4% of impurity instances)				
12.1% prevalence	84.01 ± 0.82	72.43 ± 1.54	11.58	[9.15, 14.01]
10% prevalence	80.89 ± 0.94	67.91 ± 1.68	12.98	[10.29, 15.67]
7.5% prevalence	75.55 ± 1.13	60.71 ± 1.83	14.84	[11.81, 17.87]
5% prevalence	66.73 ± 1.35	50.08 ± 1.92	16.65	[13.32, 19.97]

Each block recomputes precision under one stated departure from the test set as observed, holding every other quantity at its measured per-seed value. These are sensitivity calculations, not estimates of field precision.

Gaps are the mean of the unrounded per-seed differences, so a gap can differ by 0.01 pp from the difference of the two rounded column entries. The advantage is largest, not smallest, at field-like prevalence, because false impurity detections are penalized more heavily when impurities are rare. Re-weighting the subtype mix toward the training composition narrows the gap by about 1.5 pp at every prevalence but does not remove it. Every row favors the 3-label model in all five seeds; Cohen’s d_z_ ranges from 5.92 to 6.61.

Subtype composition. Prevalence is not the only respect in which the test set departs from the field. Soil clods make up 31.3% of its impurity instances against 12.4% in training, and the two label structures differ far more on soil clods than on stones. At the default threshold the 3-label model detects 57.3% of soil-clod instances as impurity against 35.8% for the 2-label model, a gap of 21.4 pp, whereas on stones the two are much closer (81.2% versus 77.6%, a gap of 3.6 pp). Re-weighting the impurity population to the training subtype composition therefore narrows both gaps without removing either: the IDR gap falls from 9.18 to 5.81 pp (95% CI [3.06, 8.56]) and the impurity-precision gap from 13.13 to 11.58 pp (95% CI [9.15, 14.01]), with the 3-label model still ahead in all five seeds. The two corrections act in opposite directions, and the prevalence correction is the larger of the two: applying both, at the training subtype composition and a field-realistic 5% impurity prevalence, gives a precision gap of 16.65 pp (95% CI [13.32, 19.97]). **Table 7** reports both blocks. The advantage is therefore not an artifact of the test set being soil-clod rich, although the soil-clod subtype is where most of it originates.

### 3.3 Feature-space analysis

The precision deficit established in Section 3.2 is accompanied by a measurable difference in the learned representation. Five complementary views of the 512-dimensional backbone features are reported below in this order: centroid geometry, cluster separability, per-channel discrimination, channel selectivity, and a head-free linear probe, with t-SNE and PCA projections for qualitative context.

**Table 8** reports every feature-space quantity on the same potato-versus-impurity partition. The representation learned under the 2-label structure places the two categories closer together: the centroid L2 distance falls from 5.43 ± 0.39 to 3.29 ± 0.41 (−39.4%) and the centroid cosine distance from 0.157 ± 0.031 to 0.074 ± 0.014 (−53.1%). The 5.43 for the 3-label model is a pooled value: within that model the potato–stone and potato–soil-clod centroids sit 5.50 ± 0.37 and 5.76 ± 0.44 apart, and pooling the two subtypes gives a distance below their average rather than equal to it.

**Table 8:** Feature-space comparison of the two label structures on an identical potato-versus-impurity target (mean ± SD over five seeds). For the 3-label model the stone and soil-clod labels are pooled into a single impurity class before every quantity is computed, so both models are described on the same two-category partition with the same objects and the same definitions.

Quantity	3-label	2-label	Change
Centroid L2 distance	5.43 ± 0.39	3.29 ± 0.41	−39.4%
Centroid cosine distance	0.157 ± 0.031	0.074 ± 0.014	−53.1%
Silhouette (potato vs impurity)	0.069 ± 0.008	0.043 ± 0.008	−36.6%
Per-channel Fisher, mean	0.108 ± 0.014	0.076 ± 0.013	−30.3%
Per-channel Fisher, peak	1.170 ± 0.099	0.688 ± 0.165	−41.2%
Per-channel Fisher, top 20 channels	0.682 ± 0.088	0.428 ± 0.083	−37.2%
Median channel selectivity |SI|, unfiltered	0.279 ± 0.038	0.189 ± 0.016	−32.3%
Linear-probe AUC (frozen backbone)	0.986 ± 0.001	0.975 ± 0.003	−1.1%
Linear-probe balanced accuracy	0.945 ± 0.003	0.922 ± 0.007	−2.4%

Every row is computed on the same 512-dimensional activations, the same test-set objects and the same binary labels, so the two columns are directly comparable. Change is the 2-label value relative to 3-label. Within the 3-label model the two impurity subtypes remain separable from potato individually (potato–stone Fisher 0.112 ± 0.014; potato–soil-clod 0.124 ± 0.015), which the 2-label model cannot express.

Cluster coherence moves the same way. The potato-versus-impurity silhouette falls from 0.069 ± 0.008 to 0.043 ± 0.008, a 36.6% reduction. The same reorganization is visible qualitatively in low-dimensional projections of the feature space (**Figures 2**, **3**).

**Figure 5:**
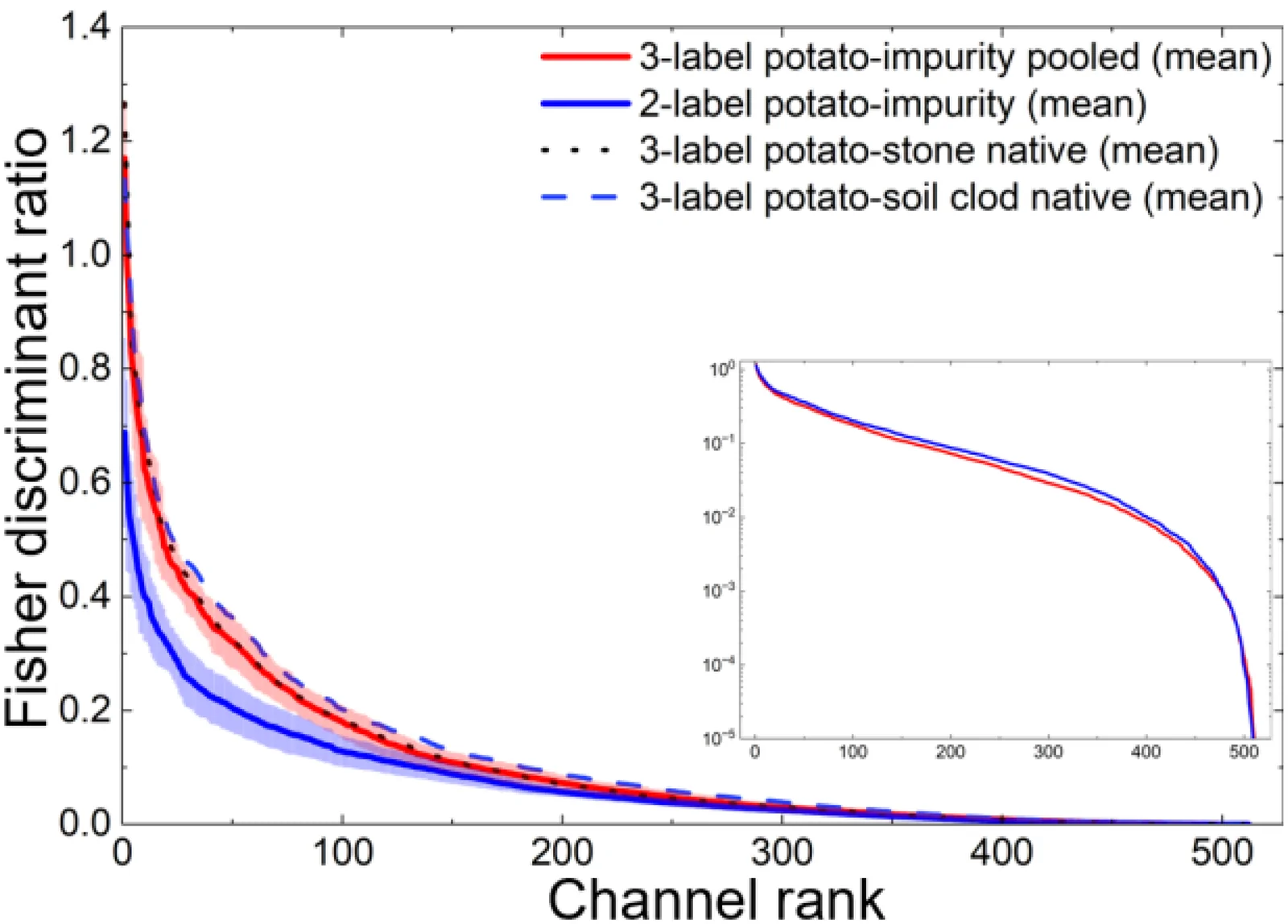
Per-channel Fisher discriminant ratio, with the 512 channels of each series sorted by its own ratio in descending order (mean ± SD over five seeds; shaded band = SD). The between-model comparison is the pair computed on the identical potato-versus-impurity partition: the 3-label curve with stone and soil clod pooled lies above the 2-label curve at 510 of 512 ranks, the two exceptions being the last two channels where both ratios are effectively zero. The inset repeats the same two curves on a logarithmic ordinate, where the separation is visible across the full channel population; the loss of discriminability under merging is therefore distributed rather than concentrated in a few channels. The two remaining curves are the 3-label model on its native subclass pairs, shown as a descriptive result only; they are not compared with the 2-label curve, which cannot express subclass structure.

At the channel level the 3-label model shows systematically higher per-channel Fisher ratios. The mean is 0.108 ± 0.014 versus 0.076 ± 0.013 (−30.3%), the peak is 1.170 ± 0.099 versus 0.688 ± 0.165 (−41.2%), and the top-20 mean is 0.682 ± 0.088 versus 0.428 ± 0.083 (−37.2%); the number of strongly discriminative channels (Fisher > 0.3) is 55 ± 11 versus 22 ± 9. **Figure 5** shows that this advantage is distributed across the channel population rather than driven by a few outliers.

**Figure 6:**
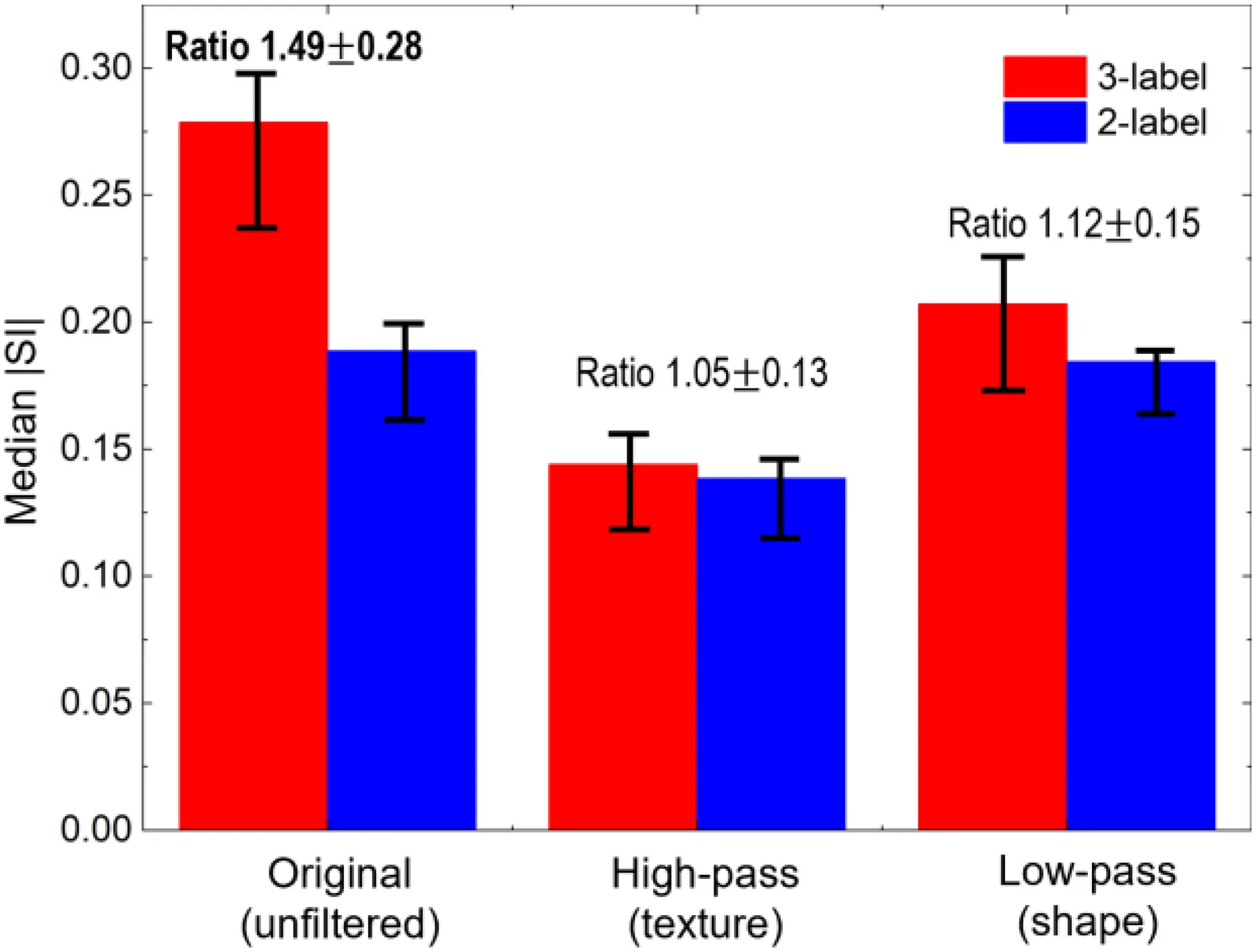
Median channel selectivity (|SI|) under unfiltered, high-pass and low-pass inputs (mean ± SD over five seeds). The 3-label advantage is present on unfiltered input (ratio 1.49 ± 0.28) but is not resolved under either filter (1.05 ± 0.13 and 1.12 ± 0.15), where the error bars overlap. The panel is therefore presented as a bounding negative result rather than as evidence of texture-specific suppression.

Channel selectivity on the unfiltered input is likewise higher for the 3-label model (median |SI| 0.279 ± 0.038 versus 0.189 ± 0.016). We additionally asked whether this selectivity advantage could be localized to a particular spatial-frequency band, by repeating the measurement on high-pass-filtered (texture-dominant) and low-pass-filtered (shape-dominant) inputs. It could not (**Figure 6**).

Finally, a frozen-backbone linear probe removes the detection head and tests separability directly. A logistic classifier on 3-label features separates potato from impurity better than on 2-label features (AUC 0.986 ± 0.001 vs. 0.975 ± 0.003; balanced accuracy 0.945 ± 0.003 vs. 0.922 ± 0.007), and under the image-level split the advantage holds down to 50 training samples per class (AUC 0.963 vs. 0.908). Because the probe bypasses the detection head, the difference is consistent with greater linear separability in the shared representation rather than with a property of the classifier. Splitting by acquisition session instead of by image lowers both models substantially and widens the seed-to-seed spread: AUC falls to 0.939 ± 0.015 for 3-label and 0.923 ± 0.011 for 2-label, and the paired advantage becomes 0.015 ± 0.022, positive in four of five seeds with a confidence interval that includes zero. Balanced accuracy behaves the same way but retains a separable advantage (0.888 ± 0.012 versus 0.856 ± 0.022; paired 0.033 ± 0.024, 95% CI [0.003, 0.062]; **Supplementary Table A4**). Session sizes are highly unequal, from a single image to 410, so a session-level split is also a higher-variance estimator than an image-level one. We therefore treat the probe as the weakest of the five views. The other four are computed without any train–test split, so no form of split leakage applies to them; the probe is the only one that requires a split, and it is the only one that loses separability under the stricter definition. The five views point the same way, but they do not carry equal weight.

**Figure 7:**
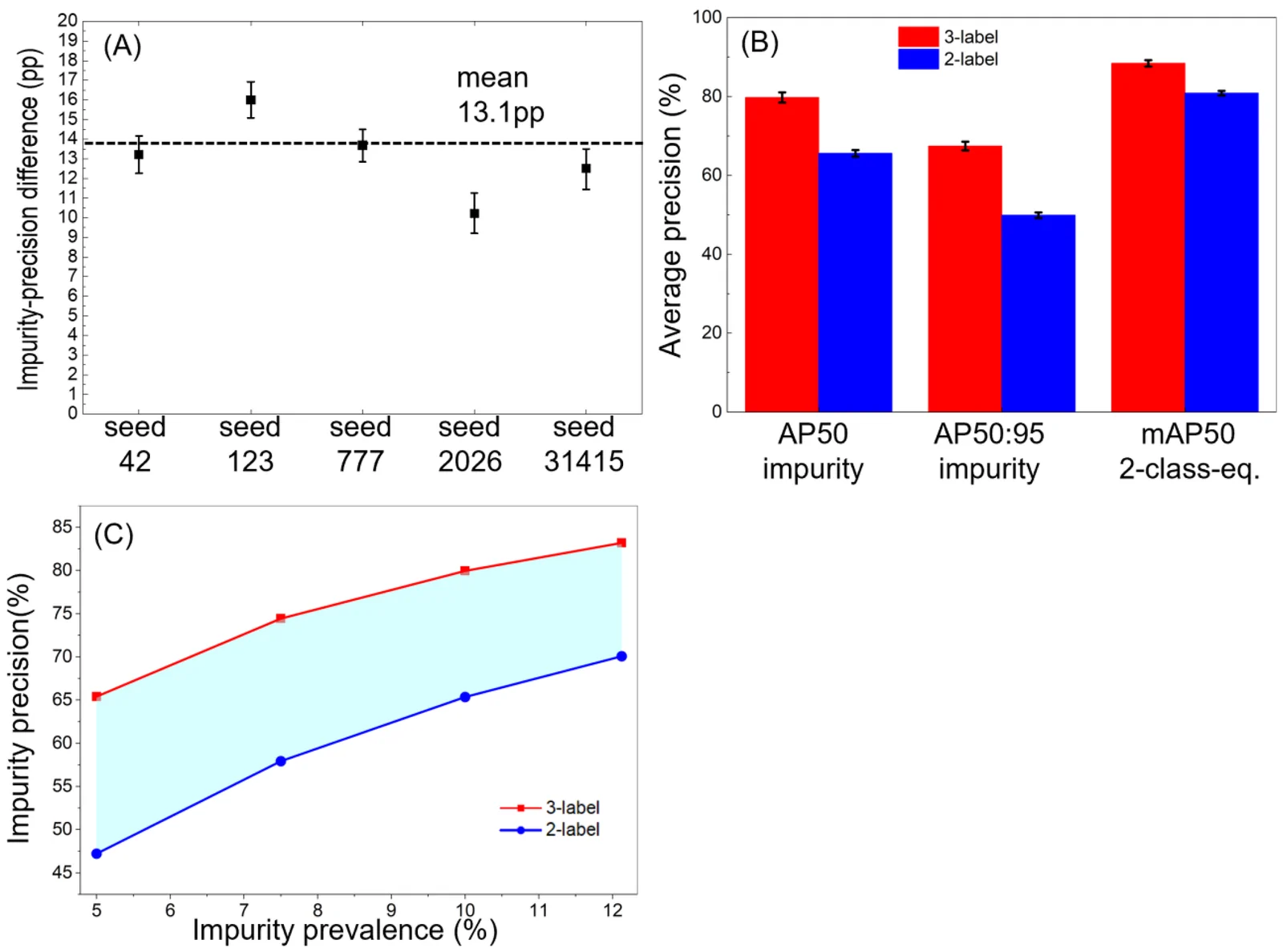
Multi-seed evidence summary. **(A)** Impurity-precision difference (3-label minus 2-label) for each of the five seeds. Error bars are within-seed bootstrap 95% confidence intervals from 10,000 resamples of the matched detections, and therefore reflect sampling uncertainty within a single training run rather than variability between runs. The dashed line is the mean across seeds, 13.13 pp; the corresponding between-seed 95% confidence interval is [10.54, 15.71] pp (Section 3.1 and **Supplementary Table A1**), and it is this wider interval that supports the inference. Every seed favors the 3-label structure. **(B)** Threshold-free average precision for both label structures (mean ± SD over five seeds). **(C)** Impurity precision re-weighted to field-realistic impurity prevalence at the observed subtype composition at the observed subtype composition, the upper block of **Table 7**; the gap widens as impurities become rarer. The rightmost point, 12.1%, is the prevalence observed in the test set.

**Figure 7** collects the multi-seed evidence that underpins Sections 3.1 and 3.2 in a single view.

## 4 Discussion

This section interprets the results and states what they do and do not establish. We first draw the feature-space evidence together, then consider why an auxiliary label structure helps even when the auxiliary task itself fails, translate the gain into field terms, and close with the limitations that bound the claim.

### 4.1 Converging evidence of a representational difference

This subsection integrates the feature-space results and states plainly how strong an inference they support. It also states, for one analysis, the interpretation that the multi-seed data does not support. Threshold analysis (Section 3.2) established that 3-label impurity precision is higher across the usable threshold landscape and across seeds. Feature analysis then offers candidate explanations at the representational level: on an identical potato-versus-impurity target, merging compresses the centroid separation (L2 −39.4%), lowers cluster coherence (silhouette −36.6%), and weakens per-channel discrimination (Fisher mean −30.3%, peak −41.2%); channel selectivity on unfiltered input is higher for 3-label, and a head-free linear probe finds 3-label features more separable. A plausible reading is that when stones and soil clods share a label, the majority subtype receives most of the impurity-classification updates by frequency and the optimizer may therefore converge on features that compress the merged class toward the potato boundary; the head-free probe is consistent with this being a property of the backbone rather than of the classification head, although it is the weakest of the five views and the only one that requires a train-test split (Section 3.3).

What the frequency decomposition does and does not support. One natural reading of the selectivity result is that merging preferentially suppresses texture-sensitive channels, which would appear as a high-pass-specific gap. Across five seeds it does not: the high-pass ratio is 1.05 ± 0.13 and the low-pass ratio is 1.12 ± 0.15, both consistent with no difference, while only the unfiltered ratio (1.49 ± 0.28) is reliably above unity. We therefore retain the frequency decomposition — it usefully bounds the mechanism — but we do not claim a texture-specific effect, and we do not describe merging as suppressing a particular class of channels. The mechanism that remains supported is the weaker and more general one: merging reduces the overall discriminability of the representation, distributed across the channel population rather than concentrated in a frequency band.

Two things must be kept apart here. The label structure was manipulated while every other element of the training setup was held constant, so both the detection difference and the representation differences are attributable to that manipulation. What is not established is the link between them: the feature-space measurements and the performance difference co-occur systematically, but we have not shown that one produces the other. A causal claim of that kind would require an interventional test, such as swapping trained backbones between detection heads, in the spirit of module-level transfer analyses (Neyshabur et al., 2020); that test remains future work.

The manipulation itself also needs one qualification. Changing the label structure changed the target semantics and the classification output dimensionality together, so the effect is attributed to the training formulation as a whole rather than to semantic granularity in isolation.

### 4.2 Auxiliary labels and feature diversity

This subsection explains how a failed subclass task can still help the primary task. The apparent paradox is that the 3-label model is poor at the task the extra label names, yet better at the task that matters. The 3-label model assigns only 1.84% of soil clods to the correct subclass yet discriminates impurity from potato more accurately. Soil clods make up just 3.3% of training instances and 12.4% of impurity instances, against stones at 87.6% — a roughly 7:1 imbalance within the merged category, so stones contribute the large majority of impurity-classification updates by frequency. We did not measure gradient magnitudes or directions, so this is a statement about example counts rather than about the optimization dynamics themselves. A resolution consistent with the multitask-learning literature (Caruana, 1997; Ruder, 2017) is that the auxiliary label structure shapes the feature space during training even when the auxiliary task itself does not succeed. The bounding-box regression and objectness terms of YOLOX’s loss act on the same objects in both label structures, so those components cannot explain the difference; whatever the auxiliary labels contribute has to enter through the subclass-specific classification target, which exists only under 3-label. That target is applied to the positive anchors assigned to soil-clod instances, and so generates backbone gradients for them even when the subclass prediction is wrong. This is our hypothesis for the route by which the label structure reaches the shared backbone; it is not measured here. The head-free linear probe (Section 3.3) is consistent with this: the 3-label representation is more linearly separable for potato-versus-impurity even though its soil-clod head is effectively non-functional. This aligns with Yang et al. (2023), who showed that coarse-label training can leave fine-grained sub-structure recoverable in the features, and with the t-SNE view (**Figure 2**) in which soil-clod samples concentrate within the stone region rather than dispersing into the potato distribution.

### 4.3 Practical consequences

This subsection translates the precision gain into field terms. The relevant cost is not accuracy in the abstract but pneumatic actuations that do no useful work. In a pneumatic removal system each false impurity detection fires a finger actuator; at 2–3 km/h, actuator fatigue, compressed-air use, and collateral displacement of nearby potatoes are the main costs of low precision. A gain of 11.6 to 13.1 percentage points in precision, depending on whether the impurity mix is left as observed or re-weighted to the training subtype composition (**Table 7**), reduces these costs without any change to the hardware. Maintaining three training labels requires no architectural change beyond one additional class channel in the classification head at each feature-pyramid level, and the annotation cost is the labor of naming a subtype the annotator is already looking at; post-processing maps both impurity subclasses to the removal action. We did not measure annotation time, inter-annotator agreement or inference latency, so we expect all three to be small, but they were not quantified. The principle may extend to other agricultural tasks in which visually similar subtypes trigger the same downstream action, such as weed species grouped for herbicide application, defect categories merged for quality grading, or pest types combined for a single intervention (Hasan et al., 2021; Liu et al., 2025), although we demonstrate it only for the present detector and crop.

### 4.4 Limitations and future directions

Several boundaries constrain how far these results should be read. We list the main ones and how the present evidence bears on each.

Replication. Results are averaged over five seeds with paired statistics; with n = 5, rank-test p-values are at their floor and effect sizes and confidence intervals carry more weight than nominal significance. Ten-seed replication is planned.

Correlation versus causation. The label-structure manipulation is experimental, so both the detection difference and the representation differences are attributable to it (Section 4.1); what remains observational is the link between them. The head-free probe is consistent with the effect residing in the representation, but it is the weakest of our five feature-space views: when the probe is held out by acquisition session rather than by image, the 3-label advantage in AUC is no longer separable from seed-to-seed variation, although it remains so in balanced accuracy and holds in four of five seeds on both metrics (**Supplementary Table A4**). The four split-free views are unaffected by this, since they involve no held-out set. Two further limitations belong here. The semantics-and-dimensionality qualification of Section 4.1 applies here too; a dummy-output or loss-normalized ablation would separate the two. And a controlled backbone-swap ablation, in the spirit of module-level transfer analyses (Neyshabur et al., 2020), is still needed for a causal claim. Both remain future work. Future work will also examine whether auxiliary loss functions or contrastive objectives (Khosla et al., 2020) can reproduce the benefit of label separation without explicit subclass annotation, and whether extending the analysis to multiscale features within the feature pyramid network (Lin et al., 2017) reveals layer-specific effects of the label structure.

Single architecture. Only YOLOX-Small was evaluated. Broad statements about agricultural detectors would be premature; whether the effect appears in Faster R-CNN (Ren et al., 2015), Transformer-based detectors (Carion et al., 2020), or newer YOLO variants (Jocher et al., 2023) is open. This includes YOLOv12, which we have benchmarked for throughput and recall but not for label structure (Kim et al., 2025). The proposed mechanism — auxiliary-label gradient diversity acting through a shared backbone — is architecture-agnostic in principle.

Split design. Training and test data are separated by harvest season and share no images or acquisition sessions, but they are drawn from overlapping farms and cultivars. The design therefore measures next-season generalization on known sites rather than generalization to unseen farms. This matches the deployment condition we care about, but it does not establish transfer to new regions; a farm-holdout or multi-region evaluation would be required for that.

Test-set construction. The test set was accumulated as difficult cases encountered by a deployed 3-label detector and is therefore not a random field sample. It is tempting to argue that this biases against the 3-label structure and makes the reported advantage conservative, but that direction cannot be established from the selection rule available to us. Cases that are hard for one detector are often intrinsically hard — small, occluded, or poorly lit objects — so the selection may act on both models similarly; but we cannot rule out that it acts asymmetrically, in either direction. Difficulty-based selection also cannot be undone after the fact; a prospectively drawn random field sample would be the clean test, and we intend it for the deployment follow-up. What the present design does support is the paired comparison, since both label structures are scored on identical images and boxes.

Regional scope. Test data are from Hokkaido. A folder-level breakdown shows the precision advantage is positive in all seven farm-named folders but varies more than fourteen-fold in magnitude (+1.6 to +23.7 pp), consistent with site-specific soil-moisture and illumination differences. Among the seven cultivar-named folders it is positive in six; the single negative value (−3.3 ± 5.0 pp) comes from one of the smallest folders, 28 images and 143 impurity instances, and its confidence interval spans zero. The folders are labeled by farm or by cultivar and do not form a farm-level partition, so this breakdown bounds folder sensitivity rather than site sensitivity. Replication in other regions would strengthen generality.

## 5 Conclusion

Across five seeds, merging two operationally equivalent impurity classes to train a YOLOX-Small potato detector lowered impurity precision at essentially every usable operating point: the gap at the default threshold is 13.1 pp, threshold-free impurity AP50 is higher by 14.2 pp, and the 3-label structure is favored in every seed across the operational region (849/849 pooled points; 5/5 seed-pairs at 100%). Five feature-space views — reduced inter-class distance, lower cluster coherence, weaker per-channel discrimination, higher channel selectivity, and a head-free linear probe — point to the same representational difference, although the linear probe is the weakest of them. Because the 3-label model derives this advantage despite collapsing the minority soil-clod subclass into stone (subclass recall 1.84 ± 0.13%, against 80.69 ± 1.12% for stones), the benefit appears attributable to the label structure itself rather than to successful subclass learning. We propose, but do not test, that it acts through the diversity of backbone gradients supplied by the auxiliary labels (Section 4.2). The representation–performance link is observational, and all results are specific to this architecture and region. For practitioners working with this class of one-stage detector, the practical implication is concrete: where multiple object types trigger the same downstream action, retaining them as separate training labels is worth testing before merging, because the merge is not free. The cost is one extra class channel per detection level and modest annotation, and the payoff is a measurably more reliable detector.

## Data Availability

The original contributions presented in the study are included in the article/**Supplementary Material**. Further inquiries can be directed to the corresponding authors.
