# Supplementary material for: Class merging degrades learned representations in potato-harvesting object detection despite operational equivalence
Source: Front Plant Sci. 2026 Sep 9;17:1847038. doi: 10.3389/fpls.2026.1847038 (PMC13597897; doi:10.3389/fpls.2026.1847038)
Supplement: Supplementary file 1 [file SupplementaryFile1.docx]

**Supplementary Material**

**Figure A1.** Representative full frames from the two corpora, at the same scale and with annotations drawn. (A), (B) 2024 corpus, from which the training and validation splits are taken. (C), (D) 2025 test set. Both corpora consist of full conveyor frames recorded from the same camera position, each containing many annotated objects; neither consists of object-centred crops. The two corpora differ in when they were recorded, not in how.

**Table A1.** Farm-composition robustness. The comparison recomputed on subsets of the test set (mean ± SD over five seeds; paired difference with 95% CI).

| **Test-set subset** | **Images** | **3-label precision (%)** | **2-label precision (%)** | **Gap**  **(pp)** | **95% CI**  **(pp)** |
| --- | --- | --- | --- | --- | --- |
| Full test set | 2,960 | 83.20 ± 0.85 | 70.07 ± 1.59 | 13.13 | [10.54, 15.71] |
| Excluding two farms | 2,325 | 83.78 ± 0.98 | 71.05 ± 1.70 | 12.73 | [9.89, 15.57] |
| Those two farms only | 635 | 80.37 ± 0.84 | 65.72 ± 1.09 | 14.65 | [12.78, 16.52] |

*The advantage is present on every subset in this table. Per-farm variation in its magnitude is discussed in Section 4.4. Cohen's d_z is 6.30, 5.56 and 9.73 respectively.*

**Table A2.** Sensitivity of the impurity-precision gap to the detection-matching IoU threshold (five seeds per setting).

| **Match IoU** | **Mean gap (pp)** | **SD (pp)** | **Min (pp)** | **Max (pp)** | **3-label higher / jointly feasible (5 × 5 subgrid)** |
| --- | --- | --- | --- | --- | --- |
| 0.30 | 12.80 | 2.11 | 9.87 | 15.74 | 118 / 118 |
| 0.50 (default) | 13.13 | 2.08 | 10.22 | 16.00 | 118 / 118 |
| 0.75 | 16.28 | 2.15 | 13.34 | 19.28 | 120 / 120 |

*The gap is positive in every seed at every setting, and grows at the stricter matching threshold. The dominance counts are computed on a coarse 5 × 5 threshold subgrid (0.1, 0.3, 0.5, 0.7, 0.9 on each axis) pooled over five seeds, not on the 17 × 17 grid of Section 3.2, because the IoU sweep was run only at those points.*

**Table A3.** Per-folder breakdown of the impurity-precision gap (mean ± SD over five seeds). The gap is 3-label minus 2-label. The fourteen folders partition the test set: seven are named by farm and seven by cultivar.

| **Test-set folder** | **Images** | **Impurity instances** | **Gap (pp)** | **SD (pp)** |
| --- | --- | --- | --- | --- |
| Kunizima (farm) | 351 | 1,466 | +23.69 | 6.39 |
| Nagaya (farm) | 214 | 3,438 | +7.06 | 2.79 |
| Ono (farm) | 102 | 393 | +1.58 | 5.21 |
| Sasakawa (farm) | 213 | 768 | +15.15 | 2.47 |
| Sato (farm) | 49 | 307 | +10.04 | 4.09 |
| Takahashi (farm) | 422 | 1,480 | +15.36 | 2.62 |
| Yamada (farm) | 455 | 2,282 | +10.34 | 3.67 |
| Dannsyaku (cultivar) | 874 | 3,594 | +11.89 | 1.08 |
| Hokkaikogane (cultivar) | 60 | 182 | +4.71 | 5.83 |
| Kitaakari (cultivar) | 105 | 928 | +23.68 | 9.93 |
| Kitahime (cultivar) | 46 | 113 | +4.52 | 7.95 |
| Konahime (cultivar) | 14 | 40 | +4.19 | 5.89 |
| Meikuin (cultivar) | 28 | 143 | −3.29 | 4.96 |
| Snowmachi (cultivar) | 27 | 100 | +12.32 | 4.65 |

*The gap is positive in all seven farm-named folders and in six of the seven cultivar-named folders. The single negative value comes from one of the smallest folders and its confidence interval spans zero. Every farm-named folder favors the 3-label model on its own. We do not claim that removing any one folder would leave the pooled result unchanged, since pooled precision is a ratio of summed counts rather than an average of folder-level rates and was not recomputed fourteen times. The folders are labeled by farm or by cultivar, so this is also not a leave-one-farm-out analysis.*

**Table A4.** Frozen-backbone linear probe under two definitions of the held-out set (mean ± SD over five seeds; the paired gap is given with its 95% confidence interval). The image-level split holds out 887 of 2,957 images; the session-level split holds out 269 of 896 acquisition sessions. All other settings are identical.

| **Split** | **Metric** | **3-label** | **2-label** | **Gap** | **95% CI** |
| --- | --- | --- | --- | --- | --- |
| Image-level | AUC | 0.986 ± 0.001 | 0.975 ± 0.003 | +0.011 ± 0.004 | [+0.007, +0.016] |
| Image-level | Balanced accuracy | 0.945 ± 0.003 | 0.922 ± 0.007 | +0.023 ± 0.009 | [+0.012, +0.034] |
| Session-level | AUC | 0.939 ± 0.015 | 0.923 ± 0.011 | +0.015 ± 0.022 | [−0.012, +0.043] |
| Session-level | Balanced accuracy | 0.888 ± 0.012 | 0.856 ± 0.022 | +0.033 ± 0.024 | [+0.003, +0.062] |

*The 3-label model is ahead in all five seeds under the image-level split and in four of five under the session-level split; the one reversal is in AUC and in balanced accuracy for the same seed. Sessions range from 1 to 410 images, so the session-level split is the higher-variance estimator. This table is the basis for treating the probe as the weakest of the five feature-space views in Section 3.3.sss*
